# Experimental study of combustion characteristics of nanoscale metal and metal oxide additives in biofuel (ethanol)

**DOI:** 10.1186/1556-276X-6-246

**Published:** 2011-03-22

**Authors:** Matthew Jones, Calvin H Li, Abdollah Afjeh, GP Peterson

**Affiliations:** 1 Department of Mechanical, Industrial, and Manufacturing Engineering University of Toledo, Toledo, OH 43606, USA; 2 Department of Mechanical Engineering, Villanova University, Villanova, PA 19085, USA; 3 The G. W. Woodruff School of Mechanical Engineering, Georgia Institute of Technology, Atlanta, GA 30332, USA

## Abstract

An experimental investigation of the combustion behavior of nano-aluminum (n-Al) and nano-aluminum oxide (n-Al_2_O_3_) particles stably suspended in biofuel (ethanol) as a secondary energy carrier was conducted. The heat of combustion (HoC) was studied using a modified static bomb calorimeter system. Combustion element composition and surface morphology were evaluated using a SEM/EDS system. N-Al and n-Al_2_O_3 _particles of 50- and 36-nm diameters, respectively, were utilized in this investigation. Combustion experiments were performed with volume fractions of 1, 3, 5, 7, and 10% for n-Al, and 0.5, 1, 3, and 5% for n-Al_2_O_3_. The results indicate that the amount of heat released from ethanol combustion increases almost linearly with n-Al concentration. N-Al volume fractions of 1 and 3% did not show enhancement in the average volumetric HoC, but higher volume fractions of 5, 7, and 10% increased the volumetric HoC by 5.82, 8.65, and 15.31%, respectively. N-Al_2_O_3 _and heavily passivated n-Al additives did not participate in combustion reactively, and there was no contribution from Al_2_O_3 _to the HoC in the tests. A combustion model that utilized Chemical Equilibrium with Applications was conducted as well and was shown to be in good agreement with the experimental results.

## Introduction

Metal additives have been utilized in solid propellants and fuels for some time and have been shown to dramatically increase combustion enthalpies and quality. In addition, these metalized propellants offer increases in the overall energy density of the fuel and increase specific impulse, and they effectively reduce the tank storage volume. In the current state-of-the-art implementation, energetic additives offer a high volumetric enthalpy of combustion, facilitating transportation of more payload per given fuel volume. However, given that the energetic additive sizes are in the micron range and sometimes even in the millimeter range, there are numerous side effects to the combustion process, including ignition delays, slow burn rates, and incomplete combustion of large (micron-sized) metal particles. Furthermore, the stability of liquid-based fuels is also a major concern; conventional liquid fuels may need to be remixed or processed before use, because of rapid settling of the energetic additive particles. New approaches and advances in nanotechnology are being developed to mitigate several of the disadvantages of metal particle additions, which will enable their large-scale implementation as viable secondary energy carriers [1].

Nanoparticle-laden fuels are known to exhibit significantly different thermophysical properties when compared to the base fuel. When metallic particles approach length scales on the order of nanometers, significant changes in thermophysical properties often occur. At these dimensions, the surface-area-to-volume ratio of the particle increases considerably, and this enables providing a larger contact surface area during the rapid oxidation process [2]. For instance, due to size-dependent properties, energetic materials containing nanoparticles can release more than twice the energy of even the best molecular explosives [3]. Several studies have reported lower melting points and lower heats of fusion for decreasing sizes of metal particles [4-6]. In particular, there are numerous combustion enhancements that result from the addition of ultrafine or nano-aluminum (n-Al) particles to gelled and solid-based propellants. Several investigators [7,8] have reported enhanced burning rates and reduced ignition delay in solid-based ammonium perchlorate propellants, in a wide array of formations. Based on these developments, research in the relatively new area of nano-energetics has become a topic of significant interest.

While there are a number of combustion enhancements resulting from the addition of nanoparticles to gelled and solid-based propellants, little investigative study has been done on the combustion properties of biofuel nanofluids. Nanoscale structures (<100 nm) stably suspended in biofuel nanofluids give rise to exciting new properties and phenomena. Previous studies have shown that the addition of nanoparticles to liquids, such as water, may improve the heat and mass transfer inside the liquid [9,10], even at low concentrations (<1 vol.%). Tyagi et al. [11] determined that adding n-Al to diesel fuel resulted in an enhancement of ignition probability when compared to the base fuel alone. With aluminum volume fractions of 0, 0.1, and 0.5%, hot plate droplets were found to have much higher ignition probability regardless of the aluminum size or form. Experimental studies with aluminum hydroxide and graphene sheets in nitromethane (NM) monopropellant resulted in significantly greater burning rates (×1.75 for graphene sheets) [12]. Likewise, nano-aluminum (n-Al)-gelling agent additives in NM resulted in increased linear and mass burning rates [13]. Suspended metallic colloids also have the ability to be optically ignited, resulting in a multipoint or "distributed ignition" within a combustion engine [14]. Experimental studies with cerium oxide fuels are known to display increased catalytic activity, causing oxidation of hydrocarbons and functioning as an oxygen buffer against NO_*x *_formation. Cerium oxide additives to biodiesel resulted in reductions of NO_*x *_by approximately 30% and reductions of hydrocarbon emissions by 25-40% [15]. Therefore, nanoparticles can function as a catalyst and an energy carrier, as well. In addition, due to the small scale of nanoparticles, the stability of the fuel suspensions should be markedly improved.

Aluminum is used due its numerous applications as an energetic material; however, current theoretical models cannot fully explain n-Al ignition in certain environmental conditions and size ranges. The phenomena of the growth of the oxide layer, effect of mechanical stresses or strains, and solid-solid phase changes or solid-liquid presence in the core are not completely understood [16]. A number of experimental investigations on aluminum additive combustion have reported a wide range of ignition temperatures even within the same particle distribution. Furthermore, the n-Al burning rate is increased with decreased particle size and is strongly dependent on temperature and pressure [17].

Previous studies have suggested that the change in oxidation temperature is triggered by metal/metalloid impurities [16], or an increasing fraction of lattice defects, or surface irregularities with decreasing particle size [4]. Trunov et al. [18] suggested that this is a result of the sequence of four polymorphic phase transformations (amorphous, γ, and α-alumina) [19], leading to a step-wise particle mass increase. In the first stage, as the metal is heated, the natural amorphous alumina layer grows until it reaches a critical thickness (approximately 5 nm), and then the oxide layer fractures and transforms into a crystalline γ-alumina phase. In the second stage, the γ-alumina oxide layer increases in density, and molten aluminum leaks through the γ-alumina faults, growing into the third stage as one of the similar intermediary transitions, such as δ or θ. In the final polymorph stage, the oxidation rate increases, and the crystalline structure becomes significantly dense as α-alumina. A qualitative analysis [18] suggested that, within the multistage oxidation, different particle self-heating rates were responsible for the range of ignition temperatures. Smaller particle ranges triggered transition to the second oxidation stage (γ-alumina) at lower temperatures; however, the transition to the second stage was delayed under higher heating rates. Rai et al. [20] proposed that aluminum nanoparticle oxidation occurs in two distinct regimes. At temperatures below the melting point of aluminum, a slow oxidation occurs with oxygen-limited diffusion through the aluminum oxide shell. At temperatures above the melting point of aluminum, a fast oxidation occurs with both aluminum and oxygen diffusing through the oxide shell, followed by a hollowing of the aluminum core at temperatures in excess of 1000°C. Recently, a new fast oxidation mechanism, referred to as the melting-dispersion mechanism, was discovered for n-Al particles under heating rates on the order of 10^7 ^C/s [17,21]. These rates are not well understood and cannot be explained by current diffusion-oxidation models. The change in volume due to fast melting of the n-Al core induces pressures in the range of 0.1-4 GPa and causes spallation of the oxide shell. As a result, further experimental studies are needed to fully characterize the n-Als as a nanoenergetic material. In this study, the combustion properties and performance of n-Al and n-Al_2_O_3 _additions to liquid ethanol (C_2_H_5_OH) are qualitatively and quantitatively investigated. Previous studies have shown a 20% increase in the thermal conductivity of ethanol with the addition of 4% volume fraction of AlN (20 nm) [22]. The primary objective of this experimental study is to characterize the combustion and gain a better understanding of n-Al oxidation in a multicomponent heterogeneous system. In order to reduce greenhouse gases from fossil-fuel use, ethanol is widely used as a biofuel and/or a fossil-fuel additive, and its complete combustion products in pure oxygen are CO_2 _and H_2_O, both of which are possible oxidizers for aluminum [23], under certain environmental conditions:(1)

Ethanol is also biodegradable and has a relatively low bio-toxicity; any spillage of pure ethanol may be simply diluted with water and disposed of down the drain [24].

Aluminum is used because of its numerous applications as an energetic material, high volumetric heat of combustion (HoC), high thermal conductivity, excellent surface absorption, and low melting/ignition temperatures. If oxygen is assumed as the primary oxidizer for aluminum combustion, then the global reaction mechanism is as follows:(2)

The main combustion product of aluminum, Al_2_O_3_, is environmentally stable and may be recycled back to pure aluminum with an electrolytic reduction [1,25]. Therefore, aluminum combustion with ethanol could potentially be regarded as a more environmentally sustainable fuel than conventional petrol if its energetic value is practical. Aluminum oxide was regarded as a heavily passivated metal and used for comparison with the ignition of pure aluminum; hence, it was hypothesized that aluminum oxide would not participate reactively in the experiments.

The nomenclature for the aluminum suspension samples will be as follows: for an aluminum nanoparticle suspension volume fraction of 5% in ethanol, it will be indicated by Eth + 5% Al, or Eth + 5% Al_2_O_3 _for alumina. The basic combustion studies here may be extended to more complex nanoenergetic systems, such as bimodal aluminum compositions, mechanically alloyed metals, or metastable intermolecular composite materials.

## Experimental setup

Combustion experiments were carried out with a modified static bomb calorimeter under a closed hood. The experiments were carried out in the presence of 2 L of distilled water with pure oxygen pressures of 20 atm. Approximately 1 g samples were placed on a stainless steel crucible, and combustion was initiated with an ignition unit via electrical discharge through a Ni-Cr alloy fuse wire (length of 10 cm) in contact with the sample. Temperature increases were determined from the average of four t-type thermocouples embedded in the system. The accuracy of the system was determined by measuring the standard energy of combustion of benzoic acid, having a quoted energy of combustion of 6318 cal/g. Using the standard procedures described in the literature [26], for ten calibrations, the experimental heat capacity for the unit was 2523.05 cal/°C. As shown in Table 1, for 15 pure ethanol runs, the experimental volumetric HoC was 21.67 ± 1.08 (MJ/L); this is in reasonable agreement with the published values. The approximated 2 MJ/L difference may be due to the use of a different grade pure ethanol in this study.

**Table 1:** Properties of ethanol fuel

Fuel	Density (g/cc)	Literature HHV (MJ/kg)	Literature HHV (MJ/L)	Experimental value (MJ/kg)	Experimental value (MJ/L)
Ethanol (99% ABV)	0.789	29.73 [24]	23.66 [24]	27.44 ± 1.35	21.67 ± 1.08

Five experiments were performed for each volume fraction and corresponding additive. N-Al and n-Al_2_O_3 _particles were of 50 and 36 nm size, respectively, as specified by the manufacturer and shown in Table 2. Both metals were suspended until they exhibited a thick and claylike consistency (i.e., to the observed threshold of nanoparticle stability). Nano-aluminum particles were suspended in pure ethanol with volumetric fractions of 1, 3, 5, 7, and 10%, and n-Al_2_O_3 _particles were suspended in pure ethanol with volumetric fractions of 0.5, 1, 3, and 5%. The total corrected enthalpies of combustion were determined from the net temperature increase and subtraction of extraneous heat of formations. For the liquid fuel samples, a fuse wire was connected to the sample by a cotton thread fuse. The cotton thread empirical formula CH_1.686_O_0.843 _was used with an energetic value of 16250 J/g [27]. Volumetric calorific values were determined from mass to volume conversions and verified by sample experimental volume measurements.

**Table 2:** Material properties of aluminum nanoparticle samples

Material	Manufacturer	Oxide shell phase	True density (g/cc)	APS (nm)	**SSA (m**^**2**^**/g)**
Al (99.9%)	Skyspring	Amorphous	2.7	50	20-48
Al_2_O_3 _(99.5%)	Nanophase	70:30, δ:γ	3.6	46	36

Samples were sonicated for at least 30 min at 47 kHz with a power rating of 143 W. Steric stabilization can be used in ethanol-based suspensions; electrostatic stabilization is often not used due to the low dielectric constant of ethanol. Previous studies of alumina powders dispersed in ethanol have shown that absorbed acetic acid (citric acid) generates a steric barrier between alumina particles [28,29]. Therefore, by modifying the acidity of the system, the suspendability can be controlled. In the current experiments, no gelling agents, apart from the nanoparticles themselves, or surfactants were used to eliminate any contribution from any additives other than nano-aluminum oxide (n-Al_2_O_3_).

**Figure 1:**
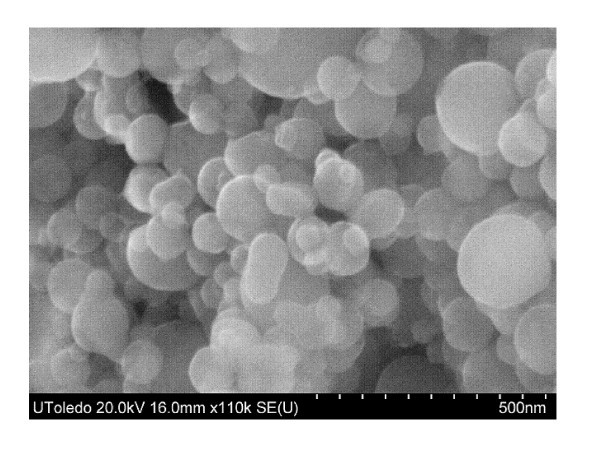
**SEM image of n-Al powder at 500 nm magnification, as received from the manufacturer**.

**Figure 2:**
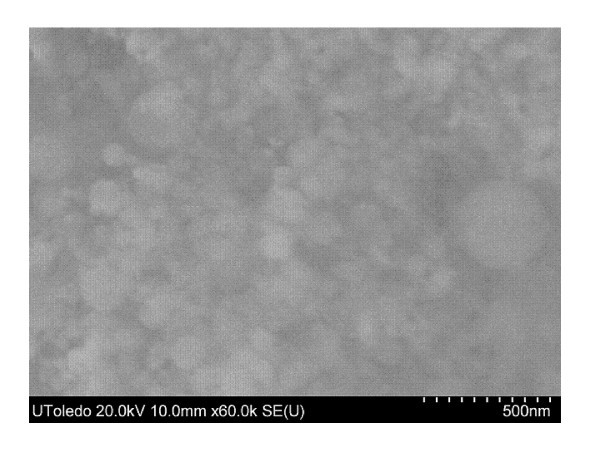
**SEM image of n-Al2O3 at 500 nm magnification, as received from the manufacturer**.

Scanning electron microscope (SEM) images in Figures 1 and 2 display the similar size diameter and size distribution of the nano-aluminum materials. An energy dispersive X-ray spectroscopy (EDS) was performed, and this resulted in an atomic composition of 78.53% Al, 19.48% O for the n-Al sample and 53.52% Al, 46.48% O for the n-Al_2_O_3 _sample. The nanoparticle material properties and fuel properties are listed in Tables 1 and 2.

**Figure 3:**
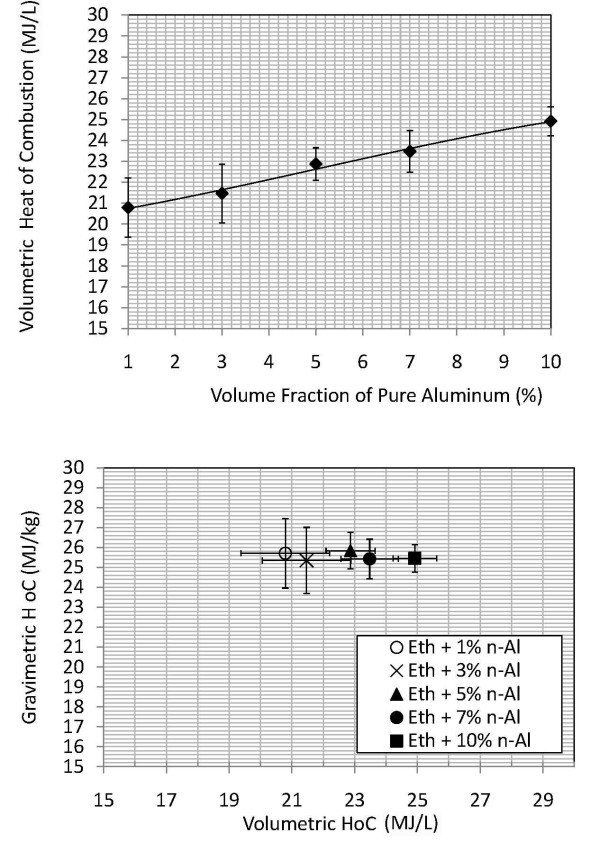
**Volumetric and gravimetric heat of combustion, ethenal with pure aluminum nanoadditives**. **(a) **Volumetric HoC of Eth + n-Al samples at 20 atm, and **(b) **volumetric and gravimetric HoC of Eth + n-Al samples.

**Figure 4:**
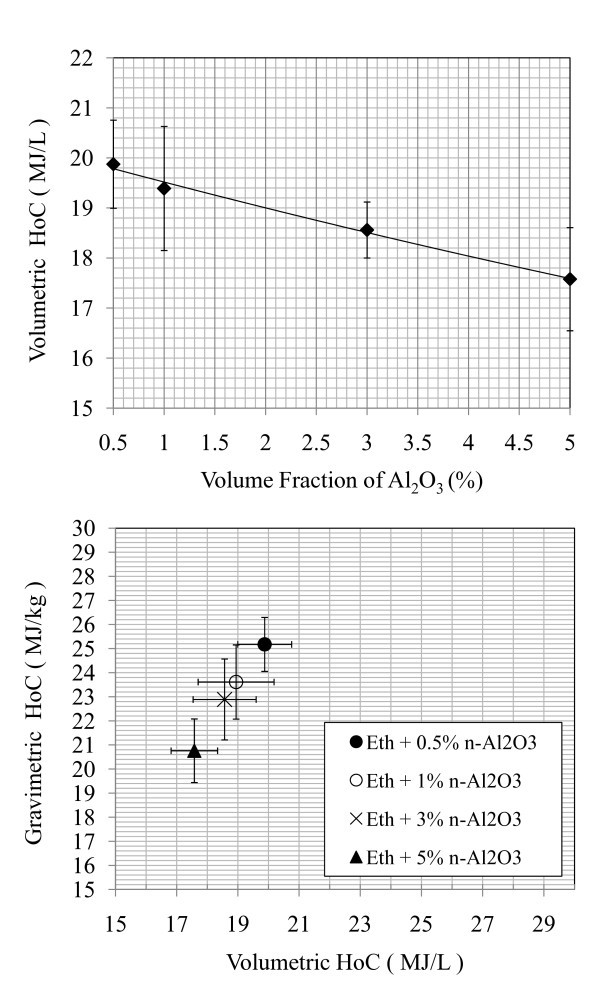
**Volume and gravimetric heat of combustion, ethenal with pure aluminum oxide nanoadditives**. **(a) **Volumetric HoC of ethanol + n-Al_2_O_3 _samples, and **(b) **volumetric and gravimetric HoC of ethanol + n-Al_2_O_3 _samples.

It is important to note that there are errors inherent to using calorimeter-type systems, despite being a well-controlled instrument to measure thermodynamic properties. The three sources of uncertainty can be attributed to the volume fraction (sample mass and volume measurements), nonadiabaticity of the system, and the performance variation of the ethanol suspensions themselves. Uncertainties in volume fraction may be inclusive to the standard error in the samples graphed in Figures 3a,b and 4a,b. A small amount of radiation may have been introduced; in this case, a radiation correction of the calorimeter is used according to ASTM Designation D240 [30]. Furthermore, the experimental calculations included in this article do not discriminate between phase change and reaction enthalpies, measuring the higher heating value (HHV) of the system. It is assumed that the entire moisture generated in the ethanol combustion has condensed. However, it may be possible that moisture generated has not fully condensed to recover the heat of vaporization given up, within the timeframe of data collection. To be conservative, an additional ±2.5% error could be added.

## Results and discussion

As shown in Figure 3a &3b, the energetic values are represented for volume fractions of Eth + n-Al samples at 1, 3, 5, 7, and 10% with a standard deviation error. Initially, at volume fractions of 1 and 3%, there was found to be a decrease in energetic release was found for n-Al ethanol suspensions. With subsequently larger volume fractions, there was an enhancement in the volumetric energy release, indicating a transition to one of the Al_2_O_3 _polymorphic phases. It was determined that the n-Al nanoparticles had an oxidized n-Al_2_O_3 _layer on the surface and that the volumetric HoC was lower than that of pure ethanol at the volume fractions of 1 and 3% due to the existence of a surface oxidization layer. Once the volume fraction was higher than 3%, more HoC was released from n-Al in the reaction process, and the volumetric HoC increased linearly. It is interesting to note that even though there was an increasing trend in HoC versus volume fraction in Figure 3a, there was a constant gravimetric HoC for all volume fractions in Figure 3b.

Figure 4a,b shows the energetic values for n-Al_2_O_3 _samples. These nanoparticles have a dominant component of Al_2_O_3 _coating that was found to increase the stability of the samples. As predicted, the n-Al_2_O_3 _nanoparticles did not react with the ambient vessel oxygen. In Figure 4b, n-Al_2_O_3 _suspensions exhibited a linear decreasing trend of energetic release because of the displacement of reactive ethanol.

**Figure 5:**
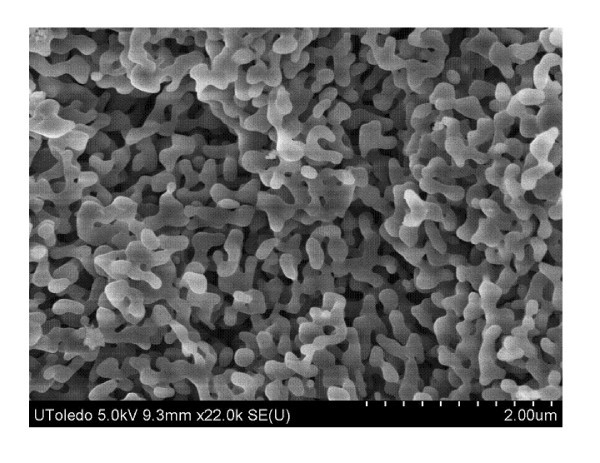
**SEM image of residual combustion products of Eth + 5% n-Al**_**2**_**O**_**3 **_**for 2.00-μm magnification**.

**Figure 6:**
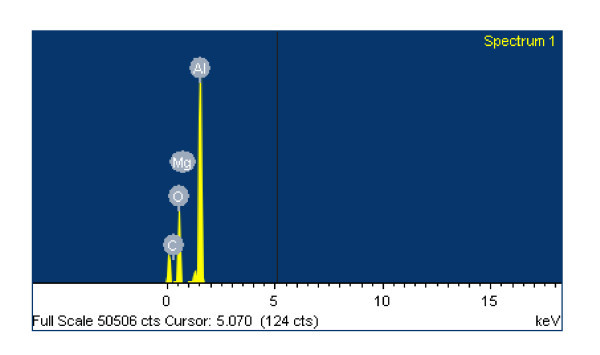
**SEM image of EDS response after combustion for Eth + 5% n-Al**.

**Figure 7:**
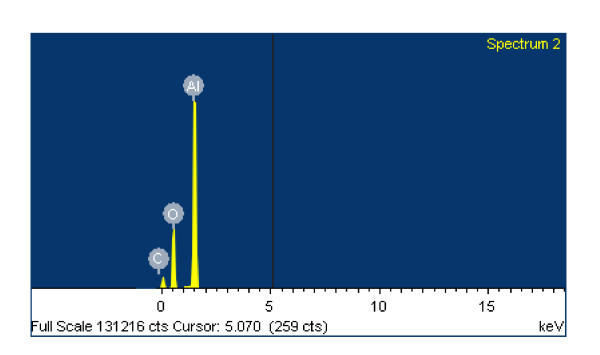
**SEM image of EDS response after combustion for Eth + 5% n-Al**_**2**_**O**_**3**_.

It was clearly illustrated in Figure 4a that the volumetric HoCs were more than 2 MJ/L lower than that of n-Al samples at equivalent volume fractions of 1 and 3%. This confirmed that the volumetric HoCs of Eth + n-Al samples at 1 and 3% were lower than that of pure ethanol due to oxidization layers. An EDS technique was performed on the residual combustion products for Eth + 5% n-Al and n-Al_2_O_3, _and it was determined that in both cases the Al:O atomic ratio was approximately 30:60, corresponding to the Al_2_O_3 _atomic composition (shown in Figures 5, 6, and 7).

Figure 5 shows the surface morphology of residual nanoparticles after combustion. It was determined that, once ignited, the nanoparticles will be quickly oxidized as n-Al_2_O_3 _and fused together. In particular, Eth + 5% n-Al samples will coagulate into droplets, while Eth + 5% n-Al_2_O_3 _will flake into a powdery substance. Furthermore, Figures 6 and 7 illustrated the near-identical EDS response after combustion, which indicates a thorough combustion of n-Al.

The experimental HoC can be determined with an energy balance on the system:(3)

where *Q *is the heat transfer between the interior of the vessel and the surrounding water, *W *is the boundary work, and *U *is the internal energy of the system. Considering a constant volume process(4)

*C*_sys _is the predetermined heat capacity of the vessel and water system, and Δ*T *is the temperature change of the system after the combustion reaction. To determine the enthalpy change within the vessel, the definition of enthalpy isused:(5)

where *H *is the enthalpy, *P *is the pressure within the vessel, and *V *is the vessel volume, 340 cm^3^. Assuming an ideal gas within the vessel, and combining Equations (4) and (5), the experimental HoC can be rewritten as(6)

where Δ*n*_gas _is the change in moles of gas of reactants and products, and *R *is the ideal gas constant. For a constant heat capacity (*C*_sys_) of the system, the final term of Equation (6) indicates that vapor products with higher flame temperatures will have larger enthalpies of combustion (HoC).

**Figure 8:**
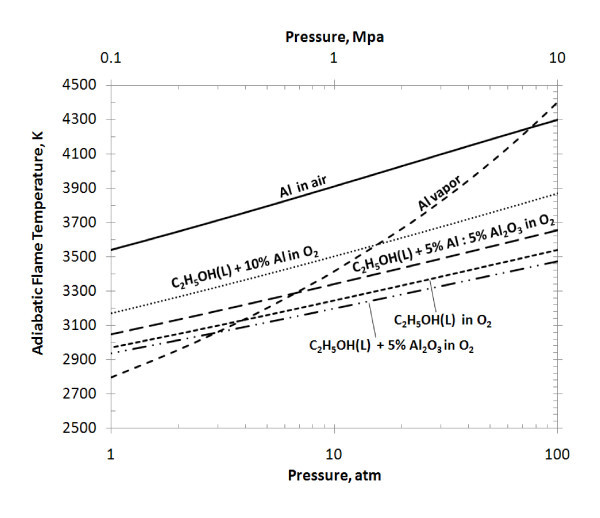
**Adiabatic flame temperatures for ethanol and aluminum mixtures at stoichiometric conditions, for an initial temperature of 298 K**.

The combustion kinetics was modeled using the NASA Chemical Equilibrium with Applications (CEA) computer program [31]. This code assumes a homogeneous system, calculates chemical equilibrium product concentrations, and determines thermodynamic properties for the product mixture. As shown in Figure 8, the calculated adiabatic flame temperatures for solid and vaporized aluminum in air were compared to liquid ethanol with Al and Al_2_O_3 _volumetric concentrations. It was assumed that all reactants were initially at room temperature (298 K). Ethanol with 10% Al concentration by volume resulted in a 6-9% increase in adiabatic flame temperature over the range of pressures' and an increase of 8.27% at the experimental 20 atm. The adiabatic flame temperature increase of 8.27% is comparable to the experimental HoC increase of 8.65% due to n-Al additives. On the other hand, ethanol with 5% Al_2_O_3 _volumetric concentration resulted in a 1-2% lower flame temperature than pure ethanol, agreeing with the experimental result that n-Al_2_O_3 _did not participate in the combustion.

The influence of the oxide layer was taken into account by incorporating a mixture of Al and Al_2_O_3 _into the fuel. For volume fractions of 5% Al + 5% Al_2_O_3 _in ethanol fuel, Figure 8 illustrates a 3.8-5.5% decrease in adiabatic flame temperature from the 10% Al ethanol mixture. This further illustrates the inert characteristics of Al_2_O_3 _and that the presence of an oxide layer significantly reduces the total combustion energy released from the Al ethanol mixture. Furthermore, it was experimentally observed that the threshold for the energetic enhancement of ethanol was with 3% volume fraction of pure Al. For the same volume fraction, the calculated CEA threshold for the flame temperature enhancement was a mixture of approximately 1.3% Al + 1.7% Al_2_O_3_. For this mixture, data processing of a 1-g sample yields 36 and 43% active Al content in mass and volume. The thickness of the oxide coating can then be estimated from the following equation [4]:(7)

where ρ_Al _(2.7 g/cc) and  (3.2 g/cc) are the Al and amorphous Al_2_O_3 _densities, *r *is the outer mean particle radius, and *c *is the pure Al content by mass. Based on the threshold of experimental and simulation energetic enhancement, the estimated oxide-layer thickness from this calculation is 6.6 nm. It is likely that the oxide-layer thickness increased because of exposure to the atmosphere during storage; additional uncertainty may be attributed to the exclusive nature of Al and Al_2_O_3 _in the software and adiabatic flame assumptions.

The change in the combustion regime may also be predicted from Figure 8. For Al and fuel-oxidizer mixtures with flame temperatures below the Al-vaporization temperature, combustion is expected to occur as a heterogeneous surface reaction, while mixtures with flame temperatures above the Al-vaporization temperature typically occur in a diffusive gas-phase. This transition in the combustion mode has been experimentally measured; a transition for 10 μm Al in oxygen was shown to occur at approximately 10 atm [32]. In Figure 8, pure ethanol in oxygen has a higher adiabatic flame temperature than the Al vaporization temperature up until approximately 4 atm. Over the same range of pressures, ethanol with 10% Al additives exhibited flame temperatures above the Al-vaporization temperature up until approximately 14 atm. This indicates that Al additives in biofuel could significantly influence the combustion regime of the mixture.

## Conclusions

Experiments have been conducted to investigate the combustion characteristics of n-Al and n-Al_2_O_3 _in ethanol. To summarize, the conclusions of this study are as follows:

1. Aluminum nanoparticles may be stably suspended in ethanol fuel up to the concentration of approximately 10% volume fraction for pure aluminum and 5% volume fraction for n-Al_2_O_3_. Although n-Al has demonstrated its ability as a gelling agent, it is recommended for future study that a dispersant is incorporated in the suspension for higher nanoparticle loadings.

2. It was experimentally shown that the amount of heat released from ethanol combustion increases almost linearly with n-Al concentrations. Nano-aluminum volume fractions of 1 and 3% deviated from the average volumetric HoC from that of pure ethanol by 3.78 and 0.66%, respectively. Higher volume fractions of 5, 7, and 10% increased the volumetric HoC by 5.82, 8.65, and 15.31%, respectively. Nano-aluminum oxide or heavily passivated n-Al does not participate reactively. Furthermore, this may be extended to other burning parameters, such as linear/mass-burning rates and ignition delay that are influenced by the amount of heat released.

3. The oxide layer has a significant effect on reaction energetics. SEM analyses and X-ray spectroscopy yielded almost identical final element compositions, despite different initial compositions. Nano-aluminum oxide displaces energetic ethanol fuel and active aluminum content, and it may function as a diffusion barrier, inhibiting phase transitions. Furthermore, thermodynamic equilibrium modeling with CEA agreed with the reaction energetics, predicting an 8.27% increase in adiabatic flame temperatures for Eth + 10% Al suspensions.

In future studies, the ignition characteristics of different nanoparticle materials in various biofuels and propellants will be investigated in various biofuels and propellants. Furthermore, future work may investigate heavier weight loadings of n-Al with the use of dispersant and identify the most effective surfactant for long-term fuel suspension stability.

## Abbreviations

EDS: energy dispersive X-ray spectroscopy; HHV: higher heating value; HoC: heat of combustion; SEM: scanning electron microscope.

## Competing interests

The authors declare that they have no competing interests.

## Authors' contributions

MJ and CHL have made substantial contributions to conception and design, acquisition of data, analysis and interpretation of data; AA and GPP have been involved in drafting the manuscript and revising it critically for important intellectual content; All the authors have given final approval of the version to be published.
